# Structure and evaluation of preventive medicine residency programs’ websites: A cross sectional analysis

**DOI:** 10.1016/j.amsu.2022.104871

**Published:** 2022-11-25

**Authors:** Maaz H. Khan, Ariba Salman, Farah Yasmin, Alina Sehar, Maham Abbasi, Shehzeen F. Memon, Muhammad Sohaib Asghar, Mohammed Mahmmoud Fadelallah Eljack, Kaleem Ullah, Muhammad Junaid Tahir

**Affiliations:** a Department of Medicine, United Medical and Dental College, Karachi, Pakistan; b Department of Medicine, Dow University of Health Sciences, Karachi, Pakistan; c Division of Nephrology and Hypertension Mayo Clinic, Rochester, Minnesota, USA; d Department of Community Medicine, Faculty of Medicine and Health Sciences, University of Bakht Alruda, Ad Duwaym, White Nile state, Sudan; e Organ Transplantation and HPB Department, Pir Abdul Qadir Shah Jeelani Institute of Medical Sciences, Gambat, Pakistan; f Department of Medicine, Lahore General Hospital, Lahore, 54000, Pakistan

**Keywords:** Preventive medicine, Website content, Residency websites, Graduate medical education, Career choice, Education

## Abstract

The goal of this study was to analyze the content availability and accessibility of preventive medicine residency program websites. In COVID-related travel restrictions, the information provided on program websites has become increasingly crucial for residency applicants.

A cross-sectional study was conducted by extracting the list of preventive medicine residency programs on the Fellowship and Residency Electronic and Interactive Database (FRIEDA). A 40-point criterion was used for the quality evaluation of residency programs. The study was conducted and analyzed in 2021.

82 preventive medicine residency programs were identified, and listed on FRIEDA, out of which 65 program websites were accessible. The median number of 40-point criteria met by the preventive medicine residency website was 25. The criteria fulfilled by the greatest number of program websites was research opportunity/facilities (94%).

The majority of the preventive medicine residency program websites were not up to the mark regarding accessibility and quality. An updated preventive medicine residency program website is essential for the applicants. Programs with relevant and precise information on their websites have higher chances of attracting potential candidates and better chances of finding the match between applicants and programs.

## Highlights

• Preventive medicine residency programs train physicians competent in public health, epidemiology, population health, and clinical medicine.
• The majority of the preventive medicine residency program websites were not up to the mark regarding accessibility and quality.
• There are no previous study on the accessibility and content of preventive medicine residency program websites.

## 1 Introduction

Preventive medicine residency programs train physicians competent in public health, epidemiology, population health, and clinical medicine [1]. Currently, it has only 82 programs out of a total of 12 417 approved graduate medical education training programs by the Accreditation Council for Graduate Medical Education (ACGME) for the current academic year (2020–2021) [2]. The scarcity of dedicated preventive medicine training programs has a ripple effect on the workforce of physicians, with only 2 preventive medicine specialists per 100 000 people in the United States (U.S.) [1] Moreover, the paucity of these training programs is partly due to the lack of industry support for preventive medicine residency programs [3]. Furthermore, a decline in desirability to pursue residency training in preventive medicine raises concerns about a sustainable workforce in the future [4]. Prior publications have discussed the reasons behind the decline of preventive medicine residency; however, website analyses for the residency programs have not been done [5,6].

There are several avenues available that medical students can utilize to obtain information about a residency program, including program websites, faculty, and program alumni [7]. In addition, professional organizations have created online databases that allow access to information such as training program demographics and statistics. Although these databases are publicly available, having an updated program website with comprehensive information about application requirements, deadlines, clinical training, didactics, and research opportunities serves as an effective educational and recruitment tool [8]. A study showed that most applicants in the interview looked upon the program web pages to decide which program to pursue. One out of seven applicants in the same study mentioned that they would rank a program on the priority that had a webpage over the one that does not [7].

Given the ease of access, websites prove to be the most versatile form of information. Therefore, several studies have shown the importance of websites for residency and fellowship applications [[9], [10], [11], [12], [13], [14], [15], [16]]. A survey of interventional radiology applicants determined program websites was an even more significant source of information for applicants than contact with residents and physician mentors [17]. The lack of in-person interviews and rotations due to the ongoing coronavirus disease 2019 (COVID-19) pandemic has further highlighted the value of an updated and well-structured post-graduate training program website [18]. Our literature search did not reveal any study on the accessibility and content of preventive medicine residency program websites. Therefore, we conducted this cross-sectional study with the aims to:

i. Evaluate the accessibility, content, and quality of ACGME accredited preventive medicine residency programs to identify potential areas of improvement.
ii. Analyze the content of the preventive medicine residency program website with different program characteristics such as geographical location and type of program.

## 2 Materials and methods

This cross-sectional study was conducted following the Strengthening the Reporting of Observational Studies in Epidemiology (STROBE) guideline.

### 2.1 Population

Fellowship and Residency Electronic and Interactive Database (FRIEDA) was used to extract a list of ACMGE-accredited preventive medicine residency programs.

### 2.2 Measure

Two independent reviewers (M.H.K and S.A.F) extracted data from FRIEDA and imported it into Microsoft Excel v.2013. All data that has been collected is accurate until July 2021. Institutional review board (IRB) approval was not needed since the information collected and analyzed in this study is publicly available. Therefore, the research was registered with local registry of Pir Abdul Qadir Shah Jeelani Institute of Medical Sciences (PAQSJIMS), Gambat, Pakistan for this study. [Reference number: IRB/21/13].

Preventive medicine residency websites were reviewed to obtain information in the following domains: 1) program overview 2) application 3) curriculum 4) current residents 5) alumni 6) faculty 7) research 8) benefits and incentives 9) residential/housing information 10) website update status.

These domains were further delineated into a 40-point criterion (Table 1) based on the variables validated in previous studies to evaluate post-graduate training program websites [10,11,15,16]. Any data available that was available regarding a specific criterion was given code "1". The data that was not available regarding a particular criterion was provided code "0". Finally, a separate 6-point scale that has previously been validated was used to evaluate the updated status of the website [11]. The criteria involved multiple factors such as 2019–2020 fellows enlisted, 2021 copyright, 2020 application deadline, and 2021 stipend information. Any website that had 3 out of 6 mentioned criteria present was considered 'updated'.

**Table 1:** 40-Point criteria breakdown showing the number of residency program websites fulfilling each criteria.

	Residency Website Criteria	Number of Programs Providing Information (n = 65)	Percentage of Programs Providing Information
1	Program Overview		
	Overview Mission Statement/Introductory Notes/Message from P.D.	58	89%
	Residency Program Director Credentials	55	85%
	Residency Program Director Contact Information	51	78%

2	Application		
	Residency Application Requirements	53	82%
	USMLE Step Scores	46	71%
	Number of Residents Recruited	38	53%
	SOPHAS Link Application	16	25%
	ERAS Link Application	28	43%
	Application Deadline	35	54%
	Interview Dates Information	42	65%

3	Curriculum		
	Didactics	54	83%
	Rotation Schedule	40	62%
	Clinical Rotation Sites	49	75%
	Prospective Residency Information	25	39%
	Associations with Societies, Organizations	23	35%
	Board Pass Rate	8	12%

4	Residents		
	Number of Residents	45	69%
	Name of Residents	45	69%
	Pictures of Residents	40	62%
	Resident's Background Information	38	59%

5	Alumni		
	Alumni Names	37	57%
	Alumni Pictures	26	40%
	Alumni Job Location	35	54%

6	Faculty		
	Faculty Name	49	75%
	Faculty Picture	48	74%
	Faculty Background Information, Credentials	44	68%

7	Research		
	Research Opportunities/Facilities	61	94%

8	Benefits and Incentives		
	Salary	38	59%
	Meal	28	43%
	Vacations	35	54%
	Educational Funds	43	66%
	Resident Wellness Program	34	52%
	Insurance	39	60%
	Visa Information	18	28%

9	Residential Information		
	Surrounding Area/Neighborhood/City Information	40	62%
	Housing Information	27	42%
	Diversity Inclusion	45	69%
	Social Media	54	83%

10	Website Updated	33	51%

Based on the type of program and geographic location, subgroup analysis was performed. Program types were defined as university-based, community-based, and military-based, as stated on FRIEDA. Four geographic regions were used to divide the program: Northeast (n = 16 [New York, Massachusetts, Connecticut, New Jersey, Pennsylvania, Maine]), South (n = 24 [Maryland, Texas, Georgia, Florida, North Carolina, Kentucky, West Virginia, Tennessee, Louisiana]), Midwest (n = 11 [Minnesota, Ohio, Illinois, Michigan, Missouri, Wisconsin]), West (n = 14 [California, Colorado, Washington, New Mexico, Utah]) (Supplemental Table 1). Data for these geographic regions were obtained from the U.S. Census Bureau regional mapping Website: www2.census.gov. The study was conducted and analyzed in 2021.

### 2.3 Statistical analysis

Based on the type of program and geographic location, we evaluated the mean number of criteria fulfilled by each program. An unpaired T-test was used to calculate the statistical significance of the mean number of criteria fulfilled by each program. Furthermore, Kruskal-Wallis H tests were performed to compare the variables based on the number of groups being compared. A p-value of <0.05 was considered significant for all cases. Statistical Product and Service Solution Software (ver. 23.0 International Business Machine Corporation, NY, USA) was used to analyze all study data.

## 3 Results

● *Numerical Distribution of Residency Programs*

**Fig. 1:**
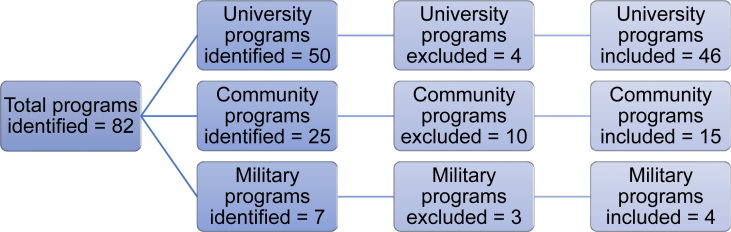
Flow chart showing the number of each type of preventive medicine program included from the total identified.

Out of 82 preventive medicine residency programs listed on FRIEDA, 65 programs fulfilled the inclusion criteria. These programs included 46 (71%) university, 15 (23%) community, and 4 (6%) military programs. Programs excluded from the analysis included 5 programs due to inaccessible links and 12 programs that had no information on their website, as shown in Fig. 1.

● *Analysis of Residency Programs Website 40-Point Criteria*

**Fig. 2:**
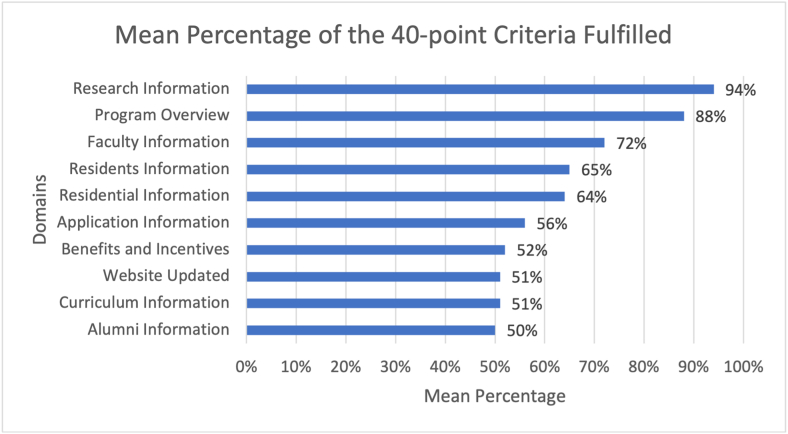
Mean percentage of the 40-point criteria fulfilled in each domain.

The median number of 40-point criteria met by the preventive medicine residency website was 25 ± 7.74 (range = 7–35). The top 5 prevalent domains to be fulfilled by each website included Research (94%), program overview (88%), faculty (72%), current residents (65%), and residential/housing information (64%), as shown in Fig. 2. However, based on the 6- point criteria applied to check for a website update, only 33(51%) of residency websites were up to date.

The percentage of highest cited criteria fulfilled by each website were research opportunities/facilities (94%), overview mission statement/introductory notes/message from Program Director (P.D.) (89%), didactics (83%), residency application requirement (82%), clinical rotation sites (75%), and faculty name (75%) as shown on Table 1. A detailed description of domains has been given below:

i. Program Overview: There were 3 criteria in this domain. It included Overview Mission Statement/Introductory Notes/Message from Program Director (58/65; 89%), Residency Program Director Credentials (55/65; 85%), Residency Program Director Contact Information (50/65; 74%).
ii. Application: Seven criteria were evaluated in this domain. The top three criteria that were fulfilled in this domain were residency application requirements (53/85; 82%), USMLE step scores (46/65; 71%), interview dates information (42/65; 65%).
iii. Curriculum: This domain had 6 criteria that were evaluated for each website. The top three criteria included were didactics (54/65; 83%), clinical rotation sites (49/65; 75%), rotation schedule (40/65; 62%).
iv. Current Residents: In this domain, 4 criteria were assessed. The top three criteria included in this domain were the number of residents (45/65; 69%), name of residents (45/65; 69%), picture of residents (40/65; 62%).
v. Alumni: Alumni is the lowest (50%) fulfilled domain. Therefore, 3 criteria were assessed in this domain. The criteria with the highest percentage of the information available included alumni names (37/65; 57%), followed by alumni job location (35/65; 54%), that was followed by alumni pictures (26/65; 40%).
vi. Faculty: This domain contained 3 out 40 criteria assessed. It included faculty name (49/65; 75%), faculty picture (48/65; 74%), faculty background information, credentials (44/65; 68%).
vii. Research: The Research was the highest (94%) fulfilled domain, and it contained 1 criteria research Opportunities/Facilities (61/65; 94%).
viii. Benefits and Incentives: Out of the 40-point criteria, this domain contained 7 criteria. The three highest criteria present in this domain were educational fund (43/65; 66%), insurance (39/65; 60%), salary (38/65; 59%).
ix. Residential/Housing Information: There were 4 criteria evaluated in this domain. The top three criteria in this domain included social media (54/65; 83%), diversity (45/65; 69%), surrounding area/neighborhood/city Information (40/65; 62%).
● *Analysis of 40-Point Criteria with Study Characteristics*

**Fig. 3:**
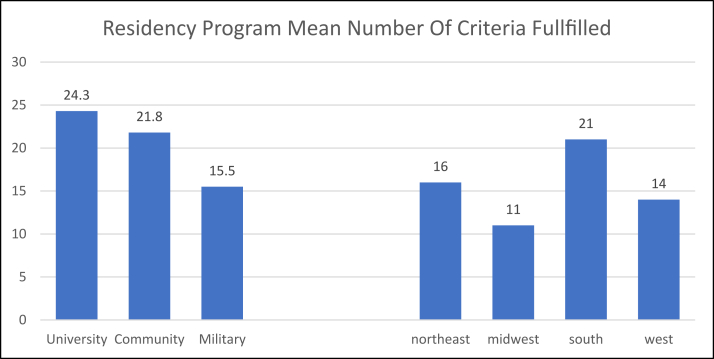
The mean number of criteria fulfilled within each type of program and region.

Fig. 3 shows the results based on the type of program and geographical location. University programs had the most criteria filled [24], whereas residency programs from the South of the U.S. had the highest score on the 40-point criteria [21]. No statistical difference was found in the mean score between the type of program and geographical location based on the 40-point criteria (p > 0.05).

## 4 Discussion

The study acts as a snapshot of the 2021 status of preventive medicine residency programs' websites, which can subsequently provide insights into why preventive medicine programs are minimal in number compared to other residency programs and face decreasing attention from prospective applicants [1,4]. Foremost, one-fifth (21%) of the original 82 programs were inaccessible due to an absent website or unavailable content on the website – a worrying sign for a specialty whose significance has been further enhanced and recognized due to the current COVID-19 pandemic [19,20]. Maintaining an accessible, informative website provides an avenue for all prospective applicants to decide where to apply, as established by Gaeta et al. for E.R. residency program websites [21].

All website domains reported 50% or greater mean percentage fulfillment as shown in Fig. 2, a satisfactory statistic showing that all programs have touched bases with the essential quality domains. In our study, program websites' most fulfilled domain was 'research' with over 94% of Preventive Medicine residency programs enlisting information regarding research opportunities on the websites. The value of research opportunities in Preventive Medicine has grown multi-fold during the current pandemic as epidemiology and infectious diseases are the two main pillars of training in the specialty. A survey by Salive et al. found graduates of the Centre of Disease Control and Prevention Preventive Medicine residency program to be the most' productive' in research [22]. Furthermore, research helps to build a base for further graduate medical education. A study on physical medicine and rehabilitation showed that physicians who have completed their residency in academic training programs and conducted research for a minimum of one year are more likely to strive for fellowship training in the future [23].

The second most fulfilled domain was "Program Overview," approaching near to 90% fulfillment; however, "Residency Program Director Contact Information" was provided by 78% of the programs, which means around less than a quarter of the programs did not provide any information to get in touch with the program director. This may be due to a reluctance to share contact information on a public website but contacting program directors through formal channels is a potential source of in-depth information for applicants. The least fulfilled domain was "Alumni Information," which was only reported by 50% of the websites that will result in applicants connecting with the graduates of only half of the programs. Alumni, alongside faculty members, can serve as important mentors for current and prospective residents regarding work-life balance, program characteristics, and future career paths [11,24].

Alarmingly, most of the programs (12%) lack information regarding board pass rates of their trainees, a finding shared by Silvestre et al. and Khan et al. as they reviewed plastic surgery residency and cardiology fellowship websites, respectively [19,24]. Board pass rates hold an essential aspect of academic credibility and quality of the program and measure its residents' performance in the board exams. Although education and didactics are a necessary facet of a residency program, resident benefits and incentives also hold importance and imply satisfactory response towards the job, as Artz demonstrates when he talks of how supportive incentives are determinants of workplace satisfaction [25]. In our study, we found that only half (52%) of the program websites mentioned information regarding benefits and incentives. Therefore, providing information regarding these basic factors will help applicants choose their choice of training program along with financial and domestic planning for the future.

International medical graduates (IMGs) are an essential addition to the U.S. health care system. Every year IMGs from around the world apply for residencies in the U.S., including 7000 non-U.S. visas requiring IMGs, according to the data provided by National Residency Match Program (NRMP) [8]. However, our study found that only 28% of program websites mentioned information about visas. Non-US IMGs are recruited into the United States health system for residency training on a J-1 exchange visitor visa or an H–1B visa [26]. Due to unforeseen circumstances, both H–1B and J1 visas can be delayed for an uncertain period. Any delay in starting the residency sanctions the training programs to waive the physician from the Match and may cause the international medical graduate to lose their job [9]. Acknowledging this obstacle and planning to improve and provide extensive information regarding visas will remove hindrances for the IMGs and help in subsequent travel planning.

We evaluated 65 programs, and none of them completely fulfilled the criteria checklist. The information provided on program websites was found inadequate for the applicants. It is recommended that the website content should be frequently monitored and updated annually at least 3–4 months before the match application process begins. Joint efforts should be initiated between the American Board of Preventive Medicine [27] and the American College of Preventive Medicine [28] to formulate a standardized checklist regarding the content on residency websites for potential applicants. Furthermore, website accessibility should be improved, and regular website checks should be done to ensure hassle-free access to the website. Addressing these deficiencies will save time and cost for the whole process, which will attract potential candidates and help recruitment in their first-choice program.

There were certain limitations to our study. First, the department or person responsible for the maintenance of the website was not contacted directly. Second, websites were solely assessed for the presence and absence of content; the quality and accuracy of the content were not analyzed. Third, we studied the website for a restricted timeframe, rather than a complete analysis that may not include changes made afterward. Fourth, some websites were inaccessible through their links, and they were not contacted any further to assess their content. Fifth, some of the programs may please provide necessary information to candidates via electronic or postal mailing methods, or maybe through online events were therefore unreachable. In the last, information gathered by candidates via any other resource was not analyzed as we included variables gleaned from Accreditation Council for Graduate Medical Education (ACGME) and some other published studies on the same topic.

## 5 Conclusion

Most U.S. Preventive Medicine residency websites lack valuable and updated information. This lack in quality indicates many opportunities are vulnerable to being missed by potential candidates. Future directions and more research work/educational awareness regarding these websites and information are necessary among medical community. Efforts to constantly improve website content and add relevant information regarding the program such as selection criteria, visa problems, incentives and benefits, alumni, and board pass rates can ensure better recruitment of applicants. Furthermore, it helps candidates make an informed decision and impact applicants’ decision to choose the program for applying, interviewing, and ranking during the match process.

The study was reviewed and approved by the Institute of Medical Sciences, GAMBAT, Pakistan's institutional review board, and was registered under the reference number IRB/21/13.

## Ethical approval

The study was reviewed and approved by the Institute of Medical Sciences, GAMBAT, Pakistan's institutional review board, and was registered under the reference number IRB/21/13.

## Sources of funding

The study was self funded.

## Authors contribution

Maaz Hasan Khan: conduct, data acquisition, data analysis, statistical analysis, manuscript preparation, manuscript editing and manuscript, final approval, and agreeing to the accuracy of the work.

Ariba Salman: planning, conduct, data acquisition, data analysis, statistical analysis, manuscript editing, and manuscript review, final approval, and agreeing to the accuracy of the work.

Alina Sehar: planning, conduct, data acquisition, data analysis, statistical analysis, manuscript editing, and manuscript review, final approval, and agreeing to the accuracy of the work.

Farah Yasmin: planning, conduct, data acquisition, data analysis, statistical analysis, manuscript editing, and manuscript review, final approval, and agreeing to the accuracy of the work editing and manuscript review, final approval, and agreeing to the accuracy of the work.

Shehzeen Fatima Memon: planning, conduct, data acquisition, data analysis, statistical analysis, manuscript editing, and manuscript review, final approval, and agreeing to the accuracy of the work editing and manuscript review, final approval, and agreeing to the accuracy of the work.

Maham Abbasi: planning, conduct, data acquisition, data analysis, statistical analysis, manuscript editing, and manuscript review, final approval, and agreeing to the accuracy of the work editing and manuscript review, final approval, and agreeing to the accuracy of the work.

Muhammad Sohaib Asghar: planning, conduct, manuscript preparation, manuscript editing and manuscript review, final approval, and agreeing to the accuracy of the work.

Mohammed Mahmmoud Fadelallah Eljack: planning, conduct, manuscript preparation, manuscript editing and manuscript review, final approval, and agreeing to the accuracy of the work.

Kaleem Ullah: manuscript editing and manuscript review, final approval, and agreeing to the accuracy of the work.

Muhammad Junaid Tahir: manuscript editing and manuscript review, final approval, and agreeing to the accuracy of the work.

## Registration of research studies

1 Name of the registry: Institute of Medical Sciences, GAMBAT, Pakistan's institutional review board
2 Unique Identifying number or registration ID: IRB/21/13.
3 Hyperlink to your specific registration (must be publicly accessible and will be checked):

## Guarantor

Muhammad Sohaib Asghar.

## Consent

N/A.

## Declaration of competing interest

Authors reports no conflict of interest.
