# Supplementary material for: Structure and evaluation of preventive medicine residency programs’ websites: A cross sectional analysis
Source: Ann Med Surg (Lond). 2022 Nov 25;84:104871. doi: 10.1016/j.amsu.2022.104871 (PMC9758379; doi:10.1016/j.amsu.2022.104871)
Supplement: Multimedia component 2 [file mmc2.docx]

**Supplemental Table 1: The Number and Type of Preventive Medicine Residency Programs in each Region and State**

| Region | State | Type of Program |  |  |  |
| --- | --- | --- | --- | --- | --- |
|  |  | University Program (n=44; 68%) | Community Program (n=17; 26%) | Military Program (n=4; 6%) | Total Number of Programs (n=65) |
| North East |  | 12 | 4 | 0 | 16 |
|  | New York | 6 | 1 | 0 | 7 |
|  | Massachusetts | 2 | 1 | 0 | 3 |
|  | Connecticut | 1 | 1 | 0 | 2 |
|  | New Jersey | 2 | 0 | 0 | 2 |
|  | Pennsylvania | 1 | 0 | 0 | 1 |
|  | Maine | 0 | 1 | 0 | 1 |
|  |  |  |  |  |  |
| South |  | 13 | 8 | 3 | 24 |
|  | Maryland | 3 | 0 | 2 | 5 |
|  | Texas | 2 | 2 | 0 | 4 |
|  | Georgia | 2 | 1 | 0 | 3 |
|  | Florida | 0 | 2 | 1 | 3 |
|  | North Carolina | 2 | 0 | 0 | 2 |
|  | Kentucky | 2 | 0 | 0 | 2 |
|  | West Virginia | 1 | 1 | 0 | 2 |
|  | Tennessee | 0 | 2 | 0 | 2 |
|  | Louisiana | 1 | 0 | 0 | 1 |
|  |  |  |  |  |  |
| Mid-West |  | 7 | 4 | 0 | 11 |
|  | Minnesota | 2 | 1 | 0 | 3 |
|  | Ohio | 1 | 1 | 0 | 2 |
|  | Illinois | 1 | 1 | 0 | 2 |
|  | Michigan | 1 | 1 | 0 | 2 |
|  | Missouri | 1 | 0 | 0 | 1 |
|  | Wisconsin | 1 | 0 | 0 | 1 |
|  |  |  |  |  |  |
| West |  | 12 | 1 | 1 | 14 |
|  | California | 8 | 0 | 0 | 8 |
|  | Colorado | 1 | 1 | 0 | 2 |
|  | Washington | 1 | 0 | 1 | 2 |
|  | New Mexico | 1 | 0 | 0 | 1 |
|  | Utah | 1 | 0 | 0 | 1 |
